# On a Variety of False Aneurism

**Published:** 1843-04

**Authors:** Robert Liston


### Selections from American and Foreign Journals.

---

*On a Variety of False Aneurism.* By ROBERT LISTON, F. R. S. Read to the Royal Medical Chirurgical Society, March, 1842.—Mr. Liston having stated, in his introductory observations, that he has met with a remarkable instance of disease, and that much benefit is likely to result from the detail of unsuccessful cases, narrates the case, first published in The Lancet, of which the following is a summary:

"A boy, nine years of age, about two months back, after an attack of cough and fever, became the subject of a swelling below the right ear, which was fomented and poulticed. The tumour slowly increased, but a few days before his admission into the hospital it began to progress rapidly. By projecting into the fauces it impeded respiration and deglutition, and it pointed near to the posterior border of the sterno-mastoid muscle. An obscure fluctuation was perceived by the touch, and some degree of pulsation in the course of the carotid artery."

Let the unprejudiced reader review this evidence, and state the nature of the case from the symptoms. Would he believe it to be one of aneurism? We ought not to credit that he did, were he to answer in the affirmative. Does he recollect the rarity of aneurism in a child? Did he ever hear of carotid aneurism in a child? Does it accord with the history of aneurism, that aneurism should appear and become developed, almost to bursting in two months? It is not usual to find the artery, in a case of true aneurism, passing over the superficial aspect of the tumour. The reverse of that is always the fact. Moreover, is it likely that an aneurism of the common carotid would acuminate behind the posterior border of the sterno-mastoid muscle? Every well educated surgeon must reply in the negative.

Mr. Liston regarded this tumour as an abscess, and introduced into it a bistoury, when out spurted a jet of arterial blood. The opening was secured, and on the following day the carotid artery was tied. In other words that treatment was adopted which would have been followed had the true natures of the tumour been detected at first. On the thirteenth day after the operation, secondary hæmorrhage from the seat of the ligature ensued. This was repeated on the fifteenth and sixteenth, and on the latter day the boy died. This treatment was accordant with sound practical surgery; and, now, what was the result of the post-mortem examination? A verification of the diagnosis of Mr. Liston. There was no disease or dilatation of the arteries, but on the posterior aspect of the common carotid, just at its bifurcation, a circular aperture was found, communicating with a cyst of condensed cellular tissue, lined by a pyogenic membrane, identical with the ordinary structure of the cyst of an abscess. The coats of the artery were seen to terminate abruptly at the edge of the opening, and there was no coagulum in the sac. This certainly was not a case of ordinary aneurism, but an instance of a rare surgical affection, an abscess communicating by ulceration with an artery, and thus giving rise to a "variety of false aneurism." The abscess had formed behind the artery, raising the latter from its bed, and separating its connections, so as to deprive it of its share of nutrient vessels. In this state of the vessel ulceration was a natural consequence; an aperture was formed, and the cyst of the abscess, previously small, became rapidly distended. We do not dwell on any question relative to the non-appearance of pus, either in the jet or after death. Who could distinguish pus, probably a small quantity, in a jet of arterial blood, or in arterial blood occupying a cyst of large size?

Having carried the matter to this point of the inquiry, we express here our opinion that Mr. Liston has conferred a benefit on medical science by recording the history of this variety. The paper is accompanied by eight cases which Mr. Liston considers to be of an analogous kind, and as proving the liability of the human system to such occurrences, and awakening attention to this additional means of forming a perfect diagnosis under similar circumstances, leading to the most judicious mode of treatment.

The first of the cases appended occurred to Dr. Craigie. It consisted in an extensive abscess of the tonsil, running down the sheath of the carotid vessels, and communicating with the common carotid, close to its bifurcation.

In the third case the femoral artery was opened, by sloughing, into a deep-seated abscess. The fourth case fell under the observation of Sir Astley Cooper.

In the fifth case, recorded by Mr. Syme, the affected artery was the popliteal. In the sixth, Mr. Gulliver's case, the carotid opened into a pharyngeal abscess. In the seventh, Mr. Fergusson's, the lingual artery communicated with an abscess in the neck; and in the eighth the opening existed in the arch of the aorta. The latter case is narrated by Breschet, and evinces much judgment and philosophy in its details.

"'It seems to prove,' says the writer, 'that medical science is thickly studded with difficulties, both as relates to diagnosis and treatment. It is to be remarked that sanguineous tumours, communicating, by a very small opening, with the interior of an artery, evince no pulsations in the early periods of the disease, when placed deeply among the tissues; and that, even in the latter stages, the movement is less a pulsation than a vibration, or trembling. This fact is deserving of attention, inasmuch as it may be the means of enabling surgeons to judge, *a priori*, whether the disease is a sanguineous tumour, produced by the dilatation of an artery, or by the *erosion* of a part of the parietes of the arterial canal.' "—*Lond. Lancet.*

